# An Efficient Human Instance-Guided Framework for Video Action Recognition

**DOI:** 10.3390/s21248309

**Published:** 2021-12-12

**Authors:** Inwoong Lee, Doyoung Kim, Dongyoon Wee, Sanghoon Lee

**Affiliations:** 1 Department of Electrical and Electronic Engineering, Yonsei University, Seoul 03722, Korea; mayddb100@yonsei.ac.kr or tnyffx@yonsei.ac.kr (D.K.); 2 Clova AI Research, NAVER Corporation, Seongnam 13561, Korea; dongyoon.wee@navercorp.com; 3 Department of Radiology, College of Medicine, Yonsei University, Seoul 03722, Korea

**Keywords:** human detection, multiple human tracking, human action recognition, convolutional neural network, temporal sequence analysis

## Abstract

In recent years, human action recognition has been studied by many computer vision researchers. Recent studies have attempted to use two-stream networks using appearance and motion features, but most of these approaches focused on clip-level video action recognition. In contrast to traditional methods which generally used entire images, we propose a new human instance-level video action recognition framework. In this framework, we represent the instance-level features using human boxes and keypoints, and our action region features are used as the inputs of the temporal action head network, which makes our framework more discriminative. We also propose novel temporal action head networks consisting of various modules, which reflect various temporal dynamics well. In the experiment, the proposed models achieve comparable performance with the state-of-the-art approaches on two challenging datasets. Furthermore, we evaluate the proposed features and networks to verify the effectiveness of them. Finally, we analyze the confusion matrix and visualize the recognized actions at human instance level when there are several people.

## 1. Introduction

Human action recognition is a highly active research area with various industrial applications including visual surveillance, video communication, gaming control and sports analysis [1,2,3,4]. Action recognition research has been mainly focused on recognition at the clip-level rather than at the human instance-level in RGB video [5,6,7,8]. With the recent development of human instance segmentation [9,10] and deep learning technology [11,12,13], human instance-level video action recognition has begun to attract considerable attention [14,15,16,17,18,19,20,21].

A human instance is defined by an individually recognized person object in an image, which can include boxes, masks and keypoints of a person. Human instance-level video action recognition uses video inputs of the human instances instead of naive cropping. Since human instance-level video action recognition requires not only distinguishing human instances from the background image but also localizing human instances, it is a very challenging research area. Because of the difficulty to obtain human instances, human instance-level video action recognition research has only recently begun to progress.

Most early video action recognition studies mainly focused on developing two-stream networks of appearance and motion features based on Convolutional Neural Networks (CNN) [5,7,22]. Since these studies were confined to clip-level processing, it was difficult to apply it into situations where multiple actions of people occur in a video. If two people in a video have different actions, it is difficult to separate the action of each person from the other at the clip-level, which inevitably fails to capture both actions. Toward an independent decision on each person, human instance-level video action recognition can provide a potential solution to resolve this issue.

There are two main issues related to human instance-level video action recognition, which are composed of how to accurately acquire human instances including metadata such as boxes, masks and keypoints, and design temporal head networks well. Specifically, the first issue also consists of three sub-issues. The first sub-issue is recognizing human instances such as boxes, masks and keypoints. This aims at distinguishing what can be subjects of actions from the background, but may be difficult when people are intertwined or there are a lot of obstacles in an image. The second sub-issue is tracking human instances. Although human instances can be separated from each image, the human instances should be linked independently throughout sequential video frames. Moreover, since human instances are not always detected in every frame of a video, it may be more difficult to make connections between temporally adjacent frames. The third sub-issue is extracting action region features defined as input features used for the temporal action head networks. The action regions can be features for human itself, and sometimes features including people, objects or surrounding backgrounds. Since action recognition performance can be severely dependent on how precisely the action region features are extracted, it is very important to represent the proper action region features. Aforementioned before, the final issue is temporal action head design related to recognizing actions using the extracted action region features. This is the most challenging issue because actions occur with lots of variations such as direction, speed and duration.

**Figure 1:**
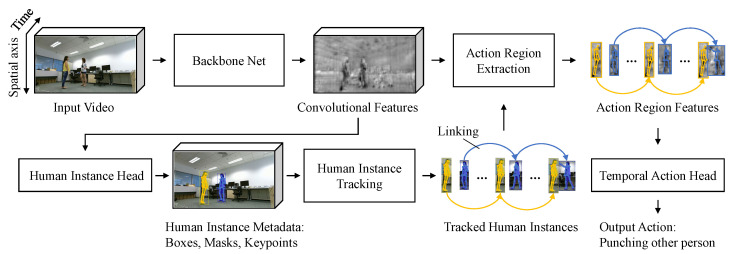
The proposed human instance-guided video action recognition framework. In the backbone network, the human instance head network is spatially done for each image in the video. Human instance tracking, action region extraction, and the temporal action head network are temporally performed for the entire video clip.

In this paper, we propose an efficient human instance-guided framework for video action recognition. Figure 1 gives an overview of our proposed framework. In contrast to the existing video action recognition models only using box data for input feature extraction, we properly use human instance metadata such as boxes, masks and keypoints for action region feature extraction and human instance tracking. Specifically, we temporally link the detected human instances obtained from backbone and human instance head network using the human keypoint metadata, and consistently extract action region features using the linked human box metadata. Since using the entire image area for action recognition involves lots of unnecessary information unrelated to recognizing actions, we only focus on interesting areas related to human actions through the extracted action region features. Unlike the existing clip-level video action recognition approaches, we individually recognize multiple actions using the temporal action head networks. The temporal action head networks increase and decrease the channel dimensions of the action region features through the various temporal action head network elements, which helps to represent action region features more effectively and capture various action dynamics well. Our main contributions are summarized as follows:

- We investigate two kinds of features such as the basic and outermost box-based action region features guided by the tracked human instance boxes. Experimentally, it is demonstrated that the proposed outermost action region features dramatically enhance the performance of action recognition.
- We propose a new type of human instance-level video action recognition framework consisting of detector, tracker and action recognizer. The detector and tracker extract the temporally connected action region features for each person, and the action recognizer determines the action using the features. In contrast to the existing works limited to clip-level recognition, our proposed models effectively recognize actions at human instance-level.
- We conduct comprehensive evaluations compared to other methods and various ablation study to demonstrate the effectiveness of our proposed model on the challenging NTU RGB + D and Northwestern ULCA Multi-view Action 3D datasets. Beyond this, we show that our proposed models work well in a variety of situations involving multiple people.

## 2. Related Work

In this section, we review the existing literature closely related to the proposed model of dealing with the issues on human instance-level video action recognition.

**Human instance recognition and tracking:** For human instance recognition, the existing object-detection research have been employed [16,17,18]. Specifically, they proposed an action proposal as the form of a tube for action localization, which was the spatio-temporal extension of Faster R-CNN only extracting boxes [14,23,24]. Unlike this approach, we propose a framework that uses human masks and keypoints as well as boxes by applying Mask R-CNN of [9] into action recognition. For human instance tracking, the authors in [16,17,18] used two criteria defined as the actionness scores and overlaps when linking the tube proposals. However, the criteria may not perform well when people cross each other because the overlap criterion just uses tube-based intersection over union between adjacent objects. Rather than using the tube-based criterion, we use the Euclidean distance between the temporally adjacent keypoints as a new criterion linking human instances.

**Action feature representation:** Video action classification, defined as recognizing what is happening in the video, has been extensively influenced by image classification research [25,26]. As a result, cropping methods used in image classification such as random crop and center crop have been also applied to video action classification. However, those cropping methods may be useful for mapping the entire video into a single action, but they are limited when it is necessary to recognize the action from each individual separately. To tackle this problem, there have been lots of studies of human instance-level video action recognition [14,15,16,17,18,19,20,21]. The authors in [14,17,19] employed the region of interest (ROI) features extracted from region proposal networks to recognize several actions. In addition to the ROI features, the authors in [15,16,18,20] crop each action region from the original RGB frame in video. Unlike these approaches, we use human instance boxes tracked by keypoint distance metric between adjacent frames and extract the outermost action regions including all ROI features in the input video clip, which helps to consistently recognize individual actions.

**Temporal action modeling:** In [27], temporal action modeling was performed by stacking the optical flows from RGB images. Then, the stacked optical flows were employed as the input features of CNNs for action recognition. In [7], each of the frames sampled from the sequence is spatio-temporally processed using the CNN model, and then the actions were recognized as averaging the spatial outputs of the CNN model. However, these studies could not individually model an action of each actor because they focused only on categorizing the videos not human instance-level detection. Early human instance-level video action recognition research modeled action by connecting regions obtained from object detectors, but they were limited to modeling action at frame-level not temporal level [14,15]. The authors of [16,17] used temporal regions from temporal proposal networks for action detection in videos, which performed better temporal modeling than the previous spatial regions. The authors of [28] used the histogram of the oriented gradient (HOG) of the Temporal Difference Map (TDMap), or frame-wise 2D CNN based on TDMap images for multiple action recognition, which is simple but vulnerable to temporal action modeling. On the other hand, Wu et al. [20] employed relatively heavy 3D backbone networks for temporal action modeling. Those 3D networks work well, but they are rather complex. In contrast to these approaches, we propose an efficient method for properly combining 2D and 3D CNN networks. We extract and link action regions related to human shapes using 2D detector, and model human instance-level actions with a 3D action recognizer. The authors of [29,30] fused the different sub-networks to reflect various action characteristics into the models, where these sub-networks were trained independently. Unlike these approaches, we share parameters of backbone and human instance head networks to learn common human instance detection, and separate the other temporal action head network parameters to learn different action characteristics, which reduces the complexity of our networks and enhances the efficiency of the networks. Furthermore, we refine the extracted action region features using the 3D convolution modules with varying channels, which enhances the channel use of the extracted action region features.

## 3. System Model

In this section, we introduce our proposed framework step by step. In addition, we present new concepts different from the existing ones in each process.

### 3.1. Human Instance Acquisition

Human instances are composed of boxes, masks and keypoints, which enable models to control human instances in the image. We use keypoints to link the temporally adjacent human instances, and also use boxes to extract action region features.

#### 3.1.1. Backbone Network

**Figure 2:**
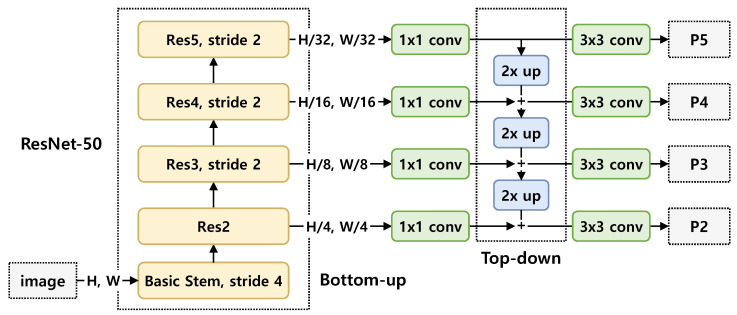
Detailed architecture of the backbone of ResNet-50-FPN. Basic Stem down-samples the input image twice by 7×7 convolution with stride 2 and max pooling with stride 2. At the first block of the res3, res4 and res5 stages, the feature map is downsampled by a convolution layer with stride 2. 2× up means 2× upsampling. The scales of P2, P3, P4 and P5 are 1/4, 1/8, 1/16 and 1/32 of the input image, respectively. The feature maps (P2, P3, P4 and P5) have 256 channels.

Backbone network is used for feature extraction over an entire image. We adopt the backbone network of Mask R-CNN [9]. Specifically, as shown in Figure 2, we use ResNet-50 [11], and use another more effective backbone network proposed by Lin et al. [31], called a Feature Pyramid Network (FPN). Our backbone network extracts the convolutional features from an entire input image, and the convolutional features are used for action region extraction. Let $g_{i}^{v}$ be the input image of the ith frame of the vth video sequence. The convolutional features of the ith frame of the vth video sequence are then obtained by

(1) $$f_{i}^{v}=\mathrm{ResNet-50-FPN}\left(g_{i}^{v}\right),\forall i\in I,\forall v\in V,$$

where *I* and *V* are the frame index set and the video index set, respectively. In (1), ResNet-50-FPN(·) means our backbone network.

#### 3.1.2. Human Instance Head Network

Human instance head network is used for bounding-box recognition, mask prediction and human pose estimation. Most of the human instance head network is almost the same as the network head of Mask R-CNN [9]. We use ResNet-50-FPN of Mask R-CNN as the head network for bounding-box recognition and mask prediction, and use the keypoint head of Mask R-CNN for human pose estimation. For further details on Mask R-CNN, we refer readers to the specific head architectures of [9].

Human instance metadata such as boxes, masks and keypoints are obtained through a combination network of the backbone network and the Human Instance Head Network (HIHNet) as follows:

(2) $$\left\{b_{i,n}^{v},m_{i,n}^{v},k_{i,n}^{v}\right\}=\mathrm{ResNet-50-FPN-HINet}\left(g_{i}^{v}\right),\forall i\in I,\forall v\in V$$

where $b_{i,n}^{v}$, $m_{i,n}^{v}$, $k_{i,n}^{v}$ denote the edge information of the box, the mask map and the keypoint/joint coordinates of the nth instance of the ith frame of the vth video sequence, respectively. In (2), ResNet-50-FPN-HINet(·) means the combination of the backbone and human instance head networks.

#### 3.1.3. Tracking Human Instances

Since the human instances are obtained independently for each frame in each video sequence, they need to be connected between frames. Let $k_{i,n,j}^{v}$ be the coordinates of the jth joint of the nth instance of the ith frame of the vth video sequence. The nth instance index of the ith frame tracked with the $n^{\prime }\mathrm{th}$ instance of the (i−1)th frame is determined by the following criterion:

(3) $$n^{*}=\underset{n\in N_{i,v}}{\arg\min}\left\{\sum_{j=0}^{J-1}\mathrm{dist}\left(k_{i,n,j}^{v},k_{i-1,n^{\prime },j}^{v}\right)\right\},\forall n^{\prime }\in N_{i-1,v}$$

where $N_{i,v}$, *J* and dist(x,y) are the instance index set of the ith frame of the vth video sequence, the total number of joints and the pixel distance between *x* and *y* points, respectively. This criterion is performed for all the frames and all the video sequences. Equation (3) indicates that the instances with the minimum joint distance between adjacent frames are regarded as the same instance. Since the tracked instance no longer depends on the frame, the *i* index of $N_{i,v}$ does not need to be used anymore.

### 3.2. Action Region Feature Extraction

**Figure 3:**
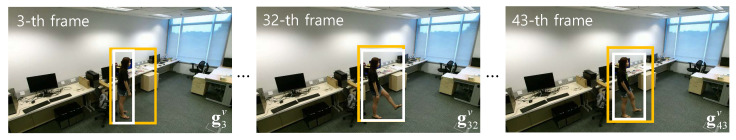
The box-based action regions of the original RGB images within an entire video. The white and yellow boxes extract the basic action region features and the action region features, respectively.

Action region represents features for action recognition, and we use box-based features as action regions. After tracking the human instances, we can extract an action region from the convolutional features through the tracked human instances. Specifically, we use the tracked box metadata among the human instances, and it is obtained by

(4) $$b_{i,n}^{v}=\left\{y_{i,n}^{L,v},x_{i,n}^{L,v},y_{i,n}^{R,v},x_{i,n}^{R,v}\right\},\forall n\in N_{v},\forall i\in I,\forall v\in V$$

where $y_{i,n}^{L,v},x_{i,n}^{L,v},y_{i,n}^{R,v},x_{i,n}^{R,v}$ are the left-top coordinates and the right-bottom coordinates of the nth box instance of the ith frame of the vth video sequence, respectively. Using the tracked box metadata, the basic box-based action region features of the nth instance of the ith frame of the vth video sequence are then obtained by

(5) $$s_{i,n}^{v}=\mathrm{ActionPooler}\left(f_{i}^{v},b_{i,n}^{v}\right),\forall n\in N_{v},\forall i\in I,\forall v\in V$$

where ActionPooler(f,b) means the ROI pooling for action recognition using the box metadata b from the convolutional features f. As shown in Figure 3, the basic action region features are extracted by the white boxes, but the sizes of the white boxes may be different each other. To make consistent spatio-temporal inputs, we use the yellow box, which is determined by the following outermost vertices of the boxes over all the frames:

(6) $${\mathrm{ob}}_{i,n}^{v}=\left\{\begin{array}{c} y_{\min,n}^{L,v}\\ x_{\min,n}^{L,v}\\ y_{\max,n}^{R,v}\\ x_{\max,n}^{R,v} \end{array}\right\}=\left\{\begin{array}{c} \min_{\forall i\in I}\left(y_{i,n}^{L,v}\right)\\ \min_{\forall i\in I}\left(x_{i,n}^{L,v}\right)\\ \max_{\forall i\in I}\left(y_{i,n}^{R,v}\right)\\ \max_{\forall i\in I}\left(x_{i,n}^{R,v}\right) \end{array}\right\},\forall n\in N_{v},\forall v\in V.$$

In contrast to the basic box-based action region features, the outermost box-based action region features of the nth instance of the ith frame of the vth video sequence are then obtained by

(7) $$r_{i,n}^{v}=\mathrm{ActionPooler}\left(f_{i}^{v},{\mathrm{ob}}_{i,n}^{v}\right),\forall n\in N_{v},\forall i\in I,\forall v\in V$$

This is performed for all the instances, all the frames and all the video sequences. In particular, the outermost box-based action region features are more consistent and give less distortion of features than the basic box-based action region features. Before the elements of (5) go through the temporal action head network, they are stacked with the frame index for the basic box-based action region features as follows:

(8) $$s_{n}^{v}=\left\{s_{i,n}^{v}\;|\;\forall i\in I\right\}.$$

Similar to this, the elements of (7) are stacked with the frame index for the outermost box-based action region features by

(9) $$r_{n}^{v}=\left\{r_{i,n}^{v}\;|\;\forall i\in I\right\}.$$

Depending on the situation, we can use either (8) or (9). In this paper, we employ the outermost box-based action region features of (9) as main action region features of the temporal action head network.

### 3.3. Temporal Action Modeling

**Figure 4:**
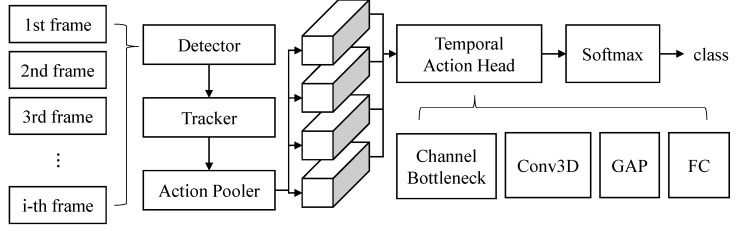
The proposed video action recognition architecture, in which the temporal action head network is composed of Channel Bottleneck (CB), Conv3D, GAP and FC. The CB module is composed of three 3D convolutions with 1×1×1 kernels that increase and decrease channels. The Conv3D module is 3D convolutions with 3×3×3 kernels that keep specific channels. GAP and FC mean global average pooling and fully connected layer, respectively.

As shown in Figure 4, the whole process of the proposed human instance-guided video action recognition framework is explained. First, each frame is processed through the detector extracting convolutional features and human instance metadata such as boxes and keypoints. Second, the acquired box metadata are tracked using the keypoint criterion. Third, each convolution feature is pooled in the action pooler using the tracked box metadata, which extracts action region features. Finally, an action label is predicted using the extracted action region features through the temporal action head network and SoftMax classifier.

#### 3.3.1. Temporal Action Head Network

Temporal action head network is used for action recognition that is applied separately to each action region feature. The action region features of the nth instance of the ith frame of the vth sequence are then processed with the Temporal Action Head Network (TAHNet) as follows:

(10) $$c_{n}^{v}=\mathrm{TAHNet}\left(s_{n}^{v}\right)$$

Similar to this, the action region features of the nth instance of the ith frame of the vth sequence are then processed with TAHNet as follows:

(11) $$d_{n}^{v}=\mathrm{TAHNet}\left(r_{n}^{v}\right)$$

As depicted in Figure 4, the temporal action head network is composed of Channel Bottleneck (CB) and Conv3D modules, GAP and FC. The CB module represents the better action region features from the convolutional features obtained by the backbone network. Through CB, the action region features are advanced by increasing and decreasing the channel dimension of the 3D convolution operations with 1×1×1 kernels. Conv3D module performs 3D convolution operations with 3×3×3 kernels that performs temporal action modeling, which strengthens the spatio-temporal modeling of the action region features. Finally, the widely used Global Average Pooling (GAP) reduces the spatio-temporal dimension to one dimension, and the reduced features are flattened and then passed through the linear FC layer.

#### 3.3.2. SoftMax Classifier and Loss Function

After the temporal action head network, the SoftMax layer value of the nth instance of the vth sequence of the basic box-based action region features is then obtained as

(12) $$\begin{array}{cc} \mathrm{Pr}(c|a_{n}^{B,v}) & =\frac{\exp(a_{n}^{B,v,c})}{\sum_{k=0}^{N_{C}-1}\exp(a_{n}^{B,v,k})}, \end{array}$$

(13) $$\begin{array}{cc} a_{n}^{B,v} & =w^{B}\cdot c_{n}^{v}+b^{B}, \end{array}$$

where *c* and $N_{C}$ are the corresponding class index and the total number of action classes, respectively. In (12), $a_{n}^{B,v}$ and $a_{n}^{B,v,k}$ are the linear activation values of all the classes and the kth class of the vth sequence in the SoftMax layer of the appearance features, respectively. In (13), $w^{B}$ and $b^{B}$ are the weight and bias terms of the SoftMax layer of the basic box-based action region features, respectively.

Similar to the basic box-based action region features, the SoftMax layer value of the nth instance of the vth sequence of the outermost box-based action region features is then obtained as

(14) $$\begin{array}{cc} \mathrm{Pr}(c|a_{n}^{O,v}) & =\frac{\exp(a_{n}^{O,v,c})}{\sum_{k=0}^{N_{C}-1}\exp(a_{n}^{O,v,k})}, \end{array}$$

(15) $$\begin{array}{cc} a_{n}^{O,v} & =w^{O}\cdot d_{n}^{v}+b^{O} \end{array}$$

where *c* and $N_{C}$ are the corresponding class index and the total number of action classes, respectively. In (14), $a_{n}^{O,v}$ and $a_{n}^{O,v,k}$ are the linear activation values of all the classes and the kth class of the vth sequence in the softmax layer of the appearance features, respectively. In (15), $w^{O}$ and $b^{O}$ are the weight and bias terms of the softmax layer of the outermost box-based action region features, respectively.

To find the maximum likelihood of all the training samples of the temporal action head network, we apply the cross-entropy function into the following objective function:

(16) $$L^{B}\;\mathrm{or}\;L^{O}=-\sum_{v=0}^{N_{V}-1}\sum_{n=0}^{N_{N}^{v}-1}\sum_{c=0}^{N_{C}-1}y_{c}^{v}\cdot \ln\left\{\mathrm{Pr}\left(c|a_{n}^{B,v}\right)\;\mathrm{or}\;\left(c|a_{n}^{O,v}\right)\right\},$$

where $y_{c}^{v}$, $N_{V}$ and $N_{N}^{v}$ are the ground-truth label of the vth sequence, the mini-batch number of training sequences and the total number of the instances of the vth sequence, respectively. We train the models by minimizing the objective function.

In the testing process, the output of the cth class of the nth instance of the vth sequence is obtained with the softmax activation value of (12) or (14). The final action classes of the nth instance of the vth sequence of the output are determined by the class indexes maximizing the value of (12) or (14).

## 4. Experimental Results

In this section, initially, we evaluate the proposed model and compare it with several recent methods on the widely used benchmark datasets: NTU RGB + D [32] and Northwestern ULCA Multi-view Action 3D dataset (N-UCLA) [33]. Next, we verify the effectiveness of the proposed methods through ablation study, and analyze the relation between actions and the temporal action head network. Finally, we show the actual use case of the proposed human instance-level action recognition model.

### 4.1. Datasets

**lNTU RGB + D dataset** [32]: This dataset was captured by 3 Microsoft Kinect v2 cameras. It is composed of 56,880 action samples including 4 different modalities of data for each sample: RGB videos, depth-map sequences, 3D skeletal data and infrared videos. It contains 60 action classes in total, which are divided into three major groups: 40 daily actions, 9 health-related actions and 11 mutual actions. It is very challenging due to the large intra-class and viewpoint variations. We follow cross-subject (CS) and cross-view (CV) evaluation protocols [32]. For the CS evaluation, half of the subjects are used for training and the remaining is used for testing on the CS evaluation. For the CV evaluation, two viewpoints are used for training, and the other is used for testing.

**N-UCLA dataset** [33]: This dataset was captured by 3 Microsoft Kinect v1 cameras. It is composed of 1,475 action samples including 3 different data modalities for each sample: RGB videos, depth-map sequences and 3D skeletal data. It contains 10 human actions performed five times by ten subjects. Each action is observed from the front, left and right views. The dataset is challenging because of varying viewpoints, self-occlusion and high similarity among actions. Since this dataset has a small amount of data, but it is rather difficult to be handled. We follow the evaluation protocol [33]. We use samples from two cameras as training data, and the samples from the rest camera as testing data.

### 4.2. Implementation Details

We use 1280×720 and 640×480 as the input video resolution for NTU RGB + D and N-UCLA, respectively. Although the original videos of 1920×1080 on the NTU RGB + D dataset improve the performance a little bit, we use the converted videos of 1280×720 as our main setting because of too long training time. For the N-UCLA dataset, we use the original video without any video converting. For data augmentation, we randomly select the temporal clip on each video during training. Since the frame lengths of each video sequence can be different from each other, we set the different frame sizes of each video sequence to a fixed frame length. Specifically, if the frame lengths of the videos are longer than the fixed frame length, then they are truncated. If the frame lengths of the videos are less than the fixed frame length, then the remaining frames are padded with 0. Each temporal clip is selected according to temporal stride, and the selected frames are used as the input of the human instance detector. All the detected instances in the same video are mapped to the same action label during training, and only one instance on each video is evaluated for testing. Specifically, the human instance with the highest instance confidence and the largest box area are selected for testing on NTU RGB + D and N-UCLA, respectively.

As previously mentioned, Mask R-CNN [9] is used for the backbone and human instance head networks and is trained by the human object labels of the COCO dataset [34]. Only human instances that are above a certain confidence threshold of 0.5 are used and tracked through the backbone and human instance head networks. When training the temporal action head network, we keep the performance of the original human detection by freezing the backbone and human instance head networks. The temporal action head network weights are learned using mini-batch stochastic gradient descent optimization. The batch is constructed by randomly selecting sequences from the training set. The batch size is set to 4 for both NTU RGB + D and N-UCLA datasets. The only difference is that 2 GPUs are used on NTU RGB + D and 1 GPU is used on N-UCLA. The learning rate is started with a value of 0.001 on both NTU RGB + D and N-ULCA. For NTU RGB + D, it is decayed by one-tenth at 80,000 and 100,000 iterations, respectively. For N-UCLA, it is maintained until the maximum iteration. The maximum iterations on NTU RGB + D and N-UCLA are 120,000 and 10,000, respectively. We use Nvidia Tesla V100-PCIE cards with 32 GB RAM as our main GPU processors. It takes from one day to four days to train the temporal action head networks using two GPUs on the NTU RGB-D dataset. Additionally, it takes from four hours to one day to train the temporal action head networks using a one GPU on the N-UCLA dataset. Since N-UCLA is small dataset, we use the models trained on the NTU RGB + D dataset, and fine-tune them on the N-UCLA dataset.

### 4.3. Comparison with SOTA

In this subsection, we compare the proposed models with the state-of-the-art methods, and these comparison methods are selected because of excellent performance on the widely used NTU RGB + D and N-UCLA datasets. Through this, we explain the difference between our models and the existing models. The proposed outermost action region features are used as the input of the proposed temporal action head network on the NTU RGB + D and N-UCLA datasets. We use four type of temporal head networks called TAHNet-v1, TAHNet-v2, TAHNet-v3 and TAHNet-v4, respectively. The specific design process is explained in Section 4.4.

As shown in Table 1, the traditional 3D pose-based methods have achieved considerable performance with a lot of research participation. With recent development of RGB video action recognition, the RGB-based methods achieve superior performance than that of the 3D pose-based methods. On the other hand, these RGB-based models have high performance for a given video clip, but it is difficult to understand how individual instances behave in the video. Although our models can continue to detect human instances by freezing the weights of the detector, we achieve the state-of-the-art performance. Specifically, TAHNet-v1 using 8 frames achieves the results (86.17%) and (89.68%) on the CS and CV evaluations, respectively, and TAHNet-v1 using 16 frames achieves the results (86.76%) and (90.14%) on the CS and CV evaluations, respectively, which are comparable with the previous RGB-based methods [35,36]. Additionally, TAHNet-v4 using 8 frames achieves the results (85.30%) and (90.02%) on the CS and CV evaluations, respectively, and TAHNet-v4 using 16 frames achieves the results (86.15%) and (90.64%) on the CS and CV evaluations, respectively. The TAHNet-v4 methods have the lowest computation complexity of (5.3 GFLOPs) and (10.6 GFLOPs), and they are comparable with the TAHNet-v4 methods. Although the performance of I3D [25] is higher than that of our models, I3D with large width and height can be quite heavy in human instance-level video action recognition application, and has more complexity of (55.9 GFLOPs). Unlike these models, our proposed models using small action region features and networks efficiently perform human instance-level video action recognition.

We follow the cross-view protocols on the N-UCLA dataset. $V_{1,2}^{3}$ means that the first and second cameras are used for training data and the third camera is used for testing data, and $V_{3,1}^{2}$ and $V_{2,3}^{1}$ are interpreted in the same way. In contrast to the NTU RGB + D dataset, the N-UCLA dataset has a small amount of data, so the overall performance is not that high as shown in Table 2. To handle the small amount of data, we use the temporal action head network trained on the NTU RGB + D dataset. The difference between pretrained and not pretrained models is at least greater than 5% in the average accuracy. Since there is considerable variation on the N-UCLA dataset, we perform training and testing processes three times per protocol to obtain consistent results, which is different from other models that overlooked the degree of deviation. Nevertheless, we achieve comparable performance with the previous RGB-based methods [35,40] using the temporal action head network. Specifically, TAHNet-v2 using 8 frames without CB and TAHNet-v2 using 16 frames without CB achieve the average accuracies of 80.6 % and 83.5 %, respectively. Additionally, the performance of the TAHNet-v2 methods are around 1% higher than those of TAHNet-v2 using 8 frames without CB and TAHNet-v2 using 16 frames without CB, which indicates that the CB modules are useful for generalization. Additionally, TAHNet-v3 using 8 frames achieves the average result (81.7%), and TAHNet-v3 using 16 frames achieves the average result (81.4%). Although the TAHNet-v3 methods have the lowest computation complexity of (25.3 GFLOPs) and (50.6 GFLOPs), they are comparable with the TAHNet-v2 methods with more complexity. Similar to NTU RGB + D, I3D [25] also has highest performance on this dataset. Since they use a large number of frames with high resolution, it is difficult to see them as the same environment.

### 4.4. Ablation Study

In this subsection, we follow the NTU-CS and UCLA-$V_{1,2}^{3}$ protocols to show the effectiveness of our proposed methods. Initially, we demonstrate the effectiveness of the proposed outermost action region features. Next, we examine the effect of each of the elements constituting the temporal action head network such as FC, Conv2D, Conv3D, and how the performance changes as Conv2D is stacked. Finally, we show the performance improvement with the addition of GAP and CB.

Table 3 shows the effects of basic box-based action region features and outermost box-based action region features. Although the basic action region features are commonly used, the outermost action region features are designed to provide consistent features to the temporal action head network. As with basic action region features, if a person is cropped every frame and resized to a fixed size, discriminative characteristics of features such as movement may be weakened. On the other hand, if the human is cropped to the outermost part of the person within the temporal window, the human movement can be expressed in a fixed space and its discriminative characteristics can be well used. The performance of the outermost action region feature is superior to that of the basic action region feature by 2.28% and 3.8% in accuracy on NTU-CS and N-UCLA, respectively, which shows that the outermost features contribute to a significant performance improvement.

Table 4 shows the effectiveness of each element in the temporal action head network. As depicted in the 2nd row, adding 2D convolution to the model using the two FC layers gives a significant improvement on both protocols. As shown in the 3rd row, the difference between Conv2D and Conv3D is 3% on the NTU-CS evaluation, which indicates that Conv3D contributes significantly to performance improvement than Conv2D. On the other hand, the performance tends to be slightly reversed on the UCLA-$V_{1,2}^{3}$ evaluation in the 2nd row. This seems to indicate that Conv2D with low complexity helps somewhat on the specific evaluation due to the small amount of data in N-UCLA. As shown in the 4th row, the accuracy tends to be saturated when the number of Conv3D is three or more on NTU-CS. The highest accuracy is achieved when the number of Conv3D is three on UCLA-$V_{1,2}^{3}$. Based on this, we use three Conv3D as the basic model for the following efficient temporal action head network design.

Table 5 shows the improvements according to the addition of GAP and the performance according to increasing the layer depth of Conv3D with CB. As depicted in the 2nd row, the addition of GAP instead of a single FC layer improves performance significantly by 3.93% and 5.1%, respectively. When increasing the layer depth of Conv3D, it has a value between 84.68% and 85.65% on the NTU-CS evaluation. On the other hand, when increasing the layer depth of Conv3D by adding CB, it has a value between 85.21% and 86.17% on the NTU-CS evaluation. Conversely, the overall performance of increasing the layer depth of Conv3D without CB is greater than that of increasing the layer depth of Conv3D with CB on the UCLA-$V_{1,2}^{3}$ evaluation. Nevertheless, we reflect the results on NTU-CS into temporal action head network design because they are more generalized in feature representation than those of UCLA-$V_{1,2}^{3}$. Based on this insight, we determine CB + $\mathrm{Conv}3D_{\times 5}$ + GAP + FC and CB + $\mathrm{Conv}3D_{\times 4}$ + GAP + FC as TAHNet-v1 and TAHNet-v2 on the NTU RGB + D and UCLA datasets, respectively.

As depicted in the 2nd row of Table 6, the action region features of CB + $\mathrm{Conv}3D_{\times 5}$ + GAP + FC (TAHNet-v1) and CB + $\mathrm{Conv}3D_{\times 4}$ + GAP + FC (TAHNet-v2) are obtained by aligning the FPN features of all scales (P2, P3, P4 and P5). As the FPN features go from P2 to P5, the FPN features are abstracted to a higher level, but they can be detector-specific features, not action-specific features. Based on this insight, we perform ablation study by removing the higher-level features in order. Overall, it can be seen that when P2 and P3 are used, the models achieve the higher performance (86.43%) and (89.6%) on the NTU-CS and UCLA-$V_{1,2}^{3}$ evaluations, respectively. This suggests that the FPN features of P2 and P3 have a common role between detector and action recognizer, and we select P2 and P3 as our FPN feature scales on both NTU RGB + D and UCLA datasets.

Table 7 shows the complexity and accuracy according to the temporal head network design. The 2nd row shows baseline models using all the FPN features of P2 and P3, and the output channel dimensions of CB are 1024, 256 and 128 in order. When changing from the 2nd row to the 3rd row, the network structure is the same, but only ${\mathrm{DIM}}_{\mathrm{Conv}}$ is reduced from 1024 to 512, which significantly reduces the complexity, but the performance is slightly lowered. When changing from the 3rd row to the 4th row, the first Conv3D kernel and stride of CB are replaced by 3 and 2 at the temporal axis, respectively. This further reduces complexity, but there is some performance drop from (1.45%) to (3.12%) on the NTU-CS evaluation. To compensate this performance drop, we increase action region feature resolution from (8×14×14) to (8×28×28). For simplicity, we remove the last linear FC layer, and change the stride of the first Conv3D from 1 to 2 at all the axes, which improves accuracy by from (1.30%) to (1.52%) on the NTU-CS evaluation. Since CB + $\mathrm{Conv}3D_{\times 3}+\mathrm{GAP}$ with the lowest complexity (5.3 GFLOPs) has adequate accuracy (85.30%), we determine the temporal head network as our TAHNet-v4 on the NTU dataset. On the other hand, the performance degradation in the 3rd and 4th rows is too severe on the UCLA-$V_{1,2}^{3}$ evaluation, so we determine CB + $\mathrm{Conv}3D_{\times 4}$ + GAP + FC with the complexity (25.3 GFLOPs) and the highest accuracy (89.8%) as our TAHNet-v3 on the UCLA dataset.

For time performance, we measure the time of detector, tracker and temporal head network as maximal frequent frame per second (fps). We use the same detector and tracker with outermost action region features, and TAHNet-v4 and TAHNet-v3 as the temporal head network on the NTU and UCLA datasets, respectively. As mentioned before, we use 8 frames with 1280×720 (padded to 1280×736) and 640×480 as the input video resolution for NTU and N-UCLA, respectively. We use Nvidia Tesla P40 cards with 24 GB RAM as time measure GPU processor. The time performance of detector, tracker and TAHNet-v4 are approximately 13 fps, 291 fps and 13.25 fps on the NTU dataset, respectively. The time performance of detector, tracker and TAHNet-v3 are approximately 22 fps, 288 fps and 7.13 fps on the NTU dataset, respectively. Specifically, the detector time performance of the NTU dataset with higher image resolution is slower that of the UCLA dataset, and the tracker time performance is similar for both datasets. The time performance of TAHNet-v4 with 5.3 GFLOPs on the NTU dataset is faster than that of TAHNet-v3 with 25.3 GFLOPs on the UCLA dataset. Although our models operate on an offline system, they have the time performance close to real-time.

### 4.5. Analysis and Visualization

**Figure 5:**
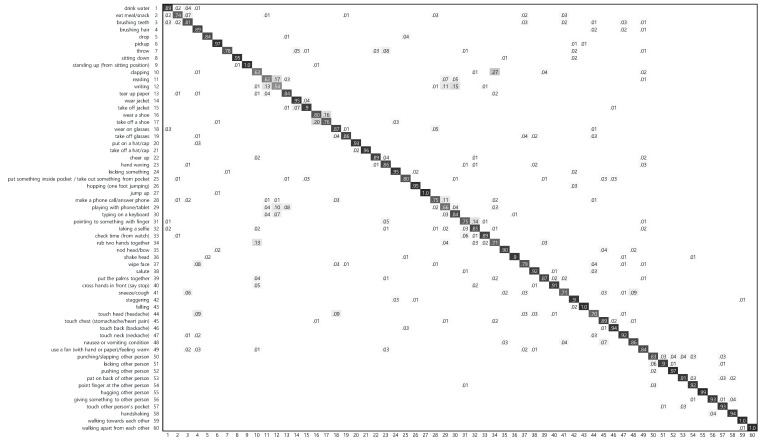
Confusion matrix of the proposed temporal action head network (TAHNet-v1) on the CS evaluation of the NTU RGB + D dataset. The overall accuracy is 86.17%.

As shown in Figure 5, the highest seven actions consist of (55) *hugging other person*, (59) *walking towards each other*, (27) *jump up*, (60) *walking apart from each other*, (6) *pickup*, (43) *falling* and (52) *pushing other person*, the accuracies of which are greater than 97%. The commonality of these actions is that they occur over a relatively large area in a consistent direction. Most of the other highest actions also have the commonality.

On the other hand, the lowest four actions are composed of (12) *writing*, (11) *reading*, (10) *clapping* and (29) *playing with phone/tablet*, the accuracies of which are lower than 70%. These actions are difficult to distinguish from the other similar actions because the other actions are generally similar in space or time, but only different in specific space or time. Specifically, (12) *writing* is very similar to (11) *reading* in the specific area. Likewise, (10) *clapping* and (29) *playing with phone/tablet* are very similar to (34) *rub two hands together* and (30) *typing on a keyboard*, respectively. This small difference between actions makes recognition difficult.

**Figure 6:**
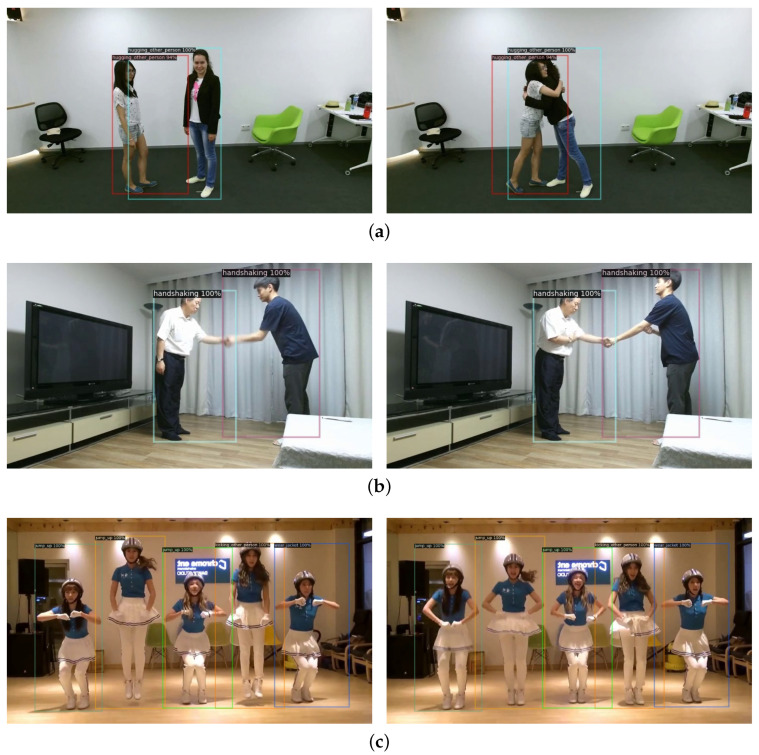
Recognized actions of the proposed human instance-level video action recognition framework. (**a**) Output frames of *hugging another person* on the NTU RGB + D dataset. (**b**) Output frames of *handshaking* on the unseen ETRI-Activity3D dataset [44,45]. (**c**) Output frames targeted by *Jump up* of in-the-wild dance video.

Figure 6 shows that the proposed human instance-level video action recognition framework visualizes the results of using video clips as input. Although most of the above-mentioned models only perform video action classification, our model accurately recognizes each action at the human instance-level. Specifically, Figure 6a shows the result of the video clip of *hugging other person* on the NTU RGB + D dataset, which works well in most cases. As shown in Figure 6b, our model also works well even when tested on the unseen dataset different from the environment in which the model was trained, *Handshaking* of the unseen ETRI-Activity3D dataset [44,45]. Beyond this case, Figure 6c shows the results of the in-the-wild dance video clip targeted by *Jump up*. The left three people are correctly detected to the action of *Jump up*, but the right two persons are misclassified to the actions of *Kicking other person* and *Wear jacket*, respectively. This is because people are too close and multiple actions are mixed within the same temporal window. Nevertheless, it is indicated that our model can also be suitable even for real environments where many people appear and move.

## 5. Conclusions

This paper addresses recognizing video actions at the human instance-level. Initially, we have acquired human instances such as boxes, masks and keypoints from the RGB videos, and connected them with each other. Next, we have extracted the action region features such as basic and outermost box-based features, and presented the efficient temporal action head networks. We have experimentally showed that the proposed models achieve comparable performance with the various state-of-the-art action recognition methods. In addition, we have performed the ablation study to verify the effectiveness of our model on the two different datasets and analyzed the relation between the classified action and the proposed method through the confusion matrix. Compared to the other models that recognize only one action in video, our model has recognized the actions of multiple people with excellent performance.

In future work, rather than addressing only video classification problem, it will be necessary to expand to human instance-level video action recognition, and to further upgrade the backbone and human instance head networks by enhancing the human instance detection accuracy using more robust 3D detector. Additionally, it will be possible to apply our model to the spatio-temporal action detection problem and other datasets with real-world environment. Our action recognition model could be used in the real-world environment, but there are still many issues such as camera movement and people being intertwined. In terms of model compression, we can refine our models to be more optimized using the lightweight deep learning techniques such as network quantization and knowledge distillation, which will enhance model inference speed and memory consumption.

## Figures and Tables

**Table 1:** Comparison results with the SOTA methods on the NTU RGB + D dataset. $N_{Clip}$ is the number of clips used for testing, respectively. *C*, *T*, *H* and *W* mean the input channel, time, height and width dimensions of the temporal action head network, respectively. GFLOPs is giga floating point operations.

Method	Pose	RGB	$N_{\mathrm{clip}}$	C×T×H×W	GFLOPs	CS	CV	Avg.
Part-aware LSTM [32]	🗸	–	–	–	–	62.93	70.27	66.6
TS-LSTM [29] (by [30])	🗸	–	–	–	–	80.07	87.25	83.66
Spatial DGNN [37]	🗸	–	–	–	–	89.2	95.5	92.4
ResNet50 + LSTM (by [35])	–	🗸	5	3×8×224×224	163.5	71.3	80.2	75.8
TCN [38]	–	🗸	1	3×20×108×192	–	80.45	82.57	81.51
Hybrid Network [36]	–	🗸	1	3×32×112×112	–	86.46	88.54	87.50
Glimpse Clouds [35]	–	🗸	5	3×8×224×224	546.5	86.6	93.2	89.9
I3D [25] (by [39])	–	🗸	1	3×32×224×224	55.9	**89.5**	**96.6**	**93.0**
TAHNet-v1	–	🗸	1	256×8×14×14	145.1	86.17	89.68	87.93
TAHNet-v1	–	🗸	1	256×16×14×14	290.3	86.76	90.14	88.45
TAHNet-v4	–	🗸	1	256×8×28×28	5.3	85.30	90.02	87.66
TAHNet-v4	–	🗸	1	256×16×28×28	10.6	86.15	90.64	88.40

**Table 2:** Comparison results with the SOTA methods on the N-UCLA dataset.

Method	Pose	RGB	$N_{\mathrm{Clip}}$	C×T×H×W	GFLOPs	$V_{1,2}^{3}$	$V_{3,1}^{2}$	$V_{2,3}^{1}$	Avg.
Enhanced vis. [41]	🗸	–	–	–	–	86.1	–	–	–
TS-LSTM [29]	🗸	–	–	–	–	89.2	–	–	–
LRCN [42]	–		1	3×16×224×224	–	–	–	–	64.7
NKTM [43]	–	🗸	–	–	–	75.8	73.3	59.1	69.4
VE-LSTM [40]	–	🗸	–	–	–	87.2	82.1	70.4	79.9
Glimpse Clouds [35]	–	🗸	5	3×8×224×224	546.5	90.1	**89.5**	**83.4**	87.6
I3D [25] (by [39])	–	🗸	1	3×32×224×224	55.9	–	–	–	**92.9**
TAHNet-v2 w/o CB	–	🗸	1	256×8×14×14	105.4	81.4±1.7	88.9±1.0	71.6±2.2	80.6
TAHNet-v2 w/o CB	–	🗸	1	256×16×14×14	210.9	84.1±1.2	89.0±2.2	77.4±1.2	83.5
TAHNet-v2	–	🗸	1	256×8×14×14	100.8	88.4±1.7	82.6±2.5	71.9±3.8	81.0
TAHNet-v2	–	🗸	1	256×16×14×14	201.5	91.7±1.4	87.0±0.7	75.5±1.0	84.7
TAHNet-v3	–	🗸	1	256×8×14×14	25.3	89.8±1.9	81.8±1.4	73.6±0.5	81.7
TAHNet-v3	–	🗸	1	256×16×14×14	50.6	88.3±0.5	84.0±2.4	71.9±5.9	81.4

**Table 3:** Experimental results according to action region feature on the NTU-CS and UCLA-$V_{1,2}^{3}$ evaluation protocols. We use TAHNet-v1 and TAHNet-v2 as the temporal action head network on NTU-CS and UCLA-$V_{1,2}^{3}$, respectively.

Feature	NTU-CS	UCLA-$V_{1,2}^{3}$
Basic action region	83.89	84.6±3.0
Outermost action region	**86.17**	88.4±1.7

**Table 4:** Experimental results according to layer type and depth. The input channel dimension of the action region features is 256, and the output channel dimensions of the FC and Conv2D layers are 1024 and 256, respectively.

Temporal Action Head	Action Region Size	NTU-CS	UCLA-$V_{1,2}^{3}$
${\mathrm{FC}}_{\times 2}$	8×7×7	69.41	73.9±1.6
$\mathrm{Conv}2D_{\times 2}$ + ${\mathrm{FC}}_{\times 2}$		74.08	80.9±1.2
$\mathrm{Conv}2D_{\times 2}$ + ${\mathrm{FC}}_{\times 2}$	8×14×14	74.82	83.6±0.3
$\mathrm{Conv}3D_{\times 2}$ + ${\mathrm{FC}}_{\times 2}$		78.44	81.7±2.0
$\mathrm{Conv}3D_{\times 1}$ + ${\mathrm{FC}}_{\times 2}$	8×14×14	75.79	80.2±2.4
$\mathrm{Conv}3D_{\times 2}$ + ${\mathrm{FC}}_{\times 2}$		78.44	81.7±2.0
$\mathrm{Conv}3D_{\times 3}$ + ${\mathrm{FC}}_{\times 2}$		80.63	83.8±1.1
$\mathrm{Conv}3D_{\times 4}$ + ${\mathrm{FC}}_{\times 2}$		80.48	81.0±7.0
$\mathrm{Conv}3D_{\times 5}$ + ${\mathrm{FC}}_{\times 2}$		**80.91**	82.8±1.0

**Table 5:** Experimental results according to the addition of network elements. The output channel dimensions of FC in all the rows, Conv3D in the 2nd row and Conv3D in the 3rd row are 1024, 256 and 1024, respectively. The output channel dimensions of CB are 1024, 256 and 128 in order.

Temporal Action Head	Action Region Size	NTU-CS	UCLA-$V_{1,2}^{3}$
$\mathrm{Conv}3D_{\times 3}$ + ${\mathrm{FC}}_{\times 2}$	8×14×14	80.63	83.8±1.1
$\mathrm{Conv}3D_{\times 3}$ + GAP + FC		84.56	88.9±2.9
$\mathrm{Conv}3D_{\times 3}$ + GAP + FC	8×14×14	84.68	88.4±0.7
$\mathrm{Conv}3D_{\times 4}$ + GAP + FC		85.65	86.4±4.1
$\mathrm{Conv}3D_{\times 5}$ + GAP + FC		85.24	88.9±1.0
$\mathrm{Conv}3D_{\times 6}$ + GAP + FC		85.38	86.4±2.4
CB + $\mathrm{Conv}3D_{\times 3}$ + GAP + FC	8×14×14	85.41	87.5±2.8
CB + $\mathrm{Conv}3D_{\times 4}$ + GAP + FC		85.48	88.4±1.7
CB + $\mathrm{Conv}3D_{\times 5}$ + GAP + FC		**86.17**	85.9±4.6
CB + $\mathrm{Conv}3D_{\times 6}$ + GAP + FC		85.21	84.9±4.4

**Table 6:** Experimental results according to the combination of FPN features.

Temporal Action Head	Action Region Size	$F_{\mathrm{FPN}}$	NTU-CS	UCLA-$V_{1,2}^{3}$
CB + $\mathrm{Conv}3D_{\times 4}$ + GAP + FC	8×14×14	P2, P3, P4, P5	85.48	88.4±1.7
CB + $\mathrm{Conv}3D_{\times 5}$ + GAP + FC			86.17	85.9±4.6
CB + $\mathrm{Conv}3D_{\times 4}$ + GAP + FC	8×14×14	P2, P3, P4	85.91	88.2±0.9
CB + $\mathrm{Conv}3D_{\times 5}$ + GAP + FC			85.60	86.8±2.0
CB + $\mathrm{Conv}3D_{\times 4}$ + GAP + FC	8×14×14	P2, P3	86.17	88.0±2.3
CB + $\mathrm{Conv}3D_{\times 5}$ + GAP + FC			**86.43**	89.6±1.3
CB + $\mathrm{Conv}3D_{\times 4}$ + GAP + FC	8×14×14	P2	86.11	88.7±1.4
CB + $\mathrm{Conv}3D_{\times 5}$ + GAP + FC			85.82	89.3±1.1

**Table 7:** Experimental results according to the network complexity. ${\mathrm{DIM}}_{\mathrm{Conv}}$ means the output channel dimension of Conv3D operation.

Temporal Action Head	${\mathrm{DIM}}_{\mathrm{Conv}}$	Action Region Size	GFLOPs	NTU-CS	UCLA-$V_{1,2}^{3}$
CB + $\mathrm{Conv}3D_{\times 3}$ + GAP + FC	1024	8×14×14→4×7×7	56.4	86.20	88.1±2.4
CB + $\mathrm{Conv}3D_{\times 4}$ + GAP + FC			100.8	86.17	88.0±2.3
CB + $\mathrm{Conv}3D_{\times 5}$ + GAP + FC			145.1	**86.43**	89.6±1.3
CB + $\mathrm{Conv}3D_{\times 6}$ + GAP + FC			189.5	86.32	86.7±1.8
CB + $\mathrm{Conv}3D_{\times 3}$ + GAP + FC	512	8×14×14→4×7×7	14.2	85.45	87.8±0.8
CB + $\mathrm{Conv}3D_{\times 4}$ + GAP + FC			25.3	85.54	89.8±1.9
CB + $\mathrm{Conv}3D_{\times 5}$ + GAP + FC			36.4	85.67	86.2±3.0
CB + $\mathrm{Conv}3D_{\times 6}$ + GAP + FC			47.5	85.89	87.8±3.7
CB + $\mathrm{Conv}3D_{\times 3}$ + GAP + FC	512	8×14×14→2×7×7	8.0	84.00	84.6±0.3
CB + $\mathrm{Conv}3D_{\times 4}$ + GAP + FC			13.5	83.60	85.1±2.8
CB + $\mathrm{Conv}3D_{\times 5}$ + GAP + FC			19.1	83.00	82.5±1.1
CB + $\mathrm{Conv}3D_{\times 6}$ + GAP + FC			24.6	82.77	81.2±6.0
CB + $\mathrm{Conv}3D_{\times 3}$ + GAP	512	8×28×28→1×7×7	5.3	85.30	81.1±2.7
CB + $\mathrm{Conv}3D_{\times 4}$ + GAP			8.1	84.92	84.8±2.9
CB + $\mathrm{Conv}3D_{\times 5}$ + GAP			10.8	84.38	82.8±0.7
CB + $\mathrm{Conv}3D_{\times 6}$ + GAP			13.6	84.29	81.4±1.4

## Data Availability

Not applicable.
